# If Tregs were people: regulation as the quiet architecture of immunity and society

**DOI:** 10.3389/fimmu.2025.1720091

**Published:** 2025-10-30

**Authors:** Bruno B. Andrade, Mariana Araújo-Pereira

**Affiliations:** ^1^ Laboratory of Clinical and Translational Research, Gonçalo Moniz Institute, Oswaldo Cruz Foundation, Salvador, Brazil; ^2^ The Biomedical Research Institute for Global Health and Translation (BRIGHT) Lab, Multinational Organization Network Sponsoring Translational and Epidemiological Research (MONSTER), Institute, Salvador, Brazil; ^3^ Disivion of Infectious Diseases, Department of Medicine, Johns Hopkins University, Baltimore, MD, United States; ^4^ Department of International Health, Bloomberg School of Public Health, Johns Hopkins University, Baltimore, MD, United States; ^5^ The Systems Biology and ImmunoLogy (SYBIL) Lab, Multinational Organization Network Sponsoring Translational and Epidemiological Research (MONSTER), Institute, Salvador, Brazil

**Keywords:** regulatory T cells, FOXP3, peripheral tolerance, IL−2, CTLA−4, adoptive cell therapy, CAR−Tregs, immunological tolerance

Regulatory T cells (Tregs) reframed immunity as a negotiated equilibrium rather than a binary of attack or retreat. This opinion argues that Tregs offer a civic metaphor for regulated disagreement: their task is not to silence immunity but to enforce context, timing, and proportion so that hosts survive conflict. Drawing on the arc from the 1990s discovery of suppressive CD4^+^CD25^+^ T cells, through the identification of FOXP3 as a lineage−defining program, to clinical strategies such as low−dose interleukin−2, adoptive Tregs, and engineered CAR−Tregs, we explore how peripheral tolerance is maintained at tissue borders and how its failure mirrors social breakdown. If Tregs were people, they would be the architects of process, mediators who convert energy into order. The lesson is practical: biological and civic systems thrive when activation is paired with rules that preserve function. Recognizing regulation as a positive capacity, rather than mere restraint, opens therapeutic and institutional horizons where diverse cells, and diverse people, can share a body.

The recognition of regulatory T cells (Tregs) as guardians of peripheral tolerance reshaped immunology. From early observations that removing CD4^+^CD25^+^ cells precipitated autoimmunity to the identification of FOXP3 as the lineage program of suppression, the field has moved from skepticism to translation (1–4). Clinically, strategies that expand or engineer Tregs now test whether calibrated regulation can restore homeostasis in inflammatory disease, transplantation, and autoimmunity (5–8).

If Tregs were people, they would not be the loudest voices, nor those whose brilliance depends on being seen first. They would arrive early, learn everyone’s names, and ensure even the fiercest debaters leave with their dignity intact. They do not shut down argument; they give it rules. Their very presence elevates the room, making people think twice before speaking. When they are absent, meetings devolve into graft rejection, collaboration into self-defense (autoimmunity), and ideas to inflammation without purpose.

A “Treg person” listen more than they speak, and when they do, it changes the rhythm of the space. They make others feel seen without seeking visibility themselves. Their steadiness is not passivity but an act of care, a commitment to preserving connection in environments that reward rupture. They do not need to win every argument; they make it possible for others to stay at the table. Tregs embody an ethic: tolerance is the presence of regulation, not the absence of response. And that, too, became a biological question.

How does an immune system so capable of violence know when to stop? The answer became one of the most profound discoveries in immunology. Tregs enforce peripheral tolerance through layered mechanisms. Tregs limit IL−2 availability by high−affinity consumption (CD25), dampen costimulation via CTLA−4, secrete anti−inflammatory cytokines such as IL−10 and TGF−β, and adopt tissue−specific programs that position them where diplomacy is most needed: at mucosal borders, in skin, and at the maternal–fetal interface (1, 3, 4). They live at the thresholds, where balance must be negotiated constantly. Without them, these borders become battlegrounds, and the body pays the price. FOXP3 maintains their identity even under inflammatory pressure, sustaining a suppressive transcriptome and regulatory circuitry - a rare ability, in cells and in people alike.

A world with less tolerance (biological or social) is a world with more damage. In the body, myocarditis after trivial provocation, colitis against commensals, thyroiditis long after the fever has passed. Socially, it resembles institutions that cannot absorb disagreement—platforms that reward outrage, laboratories that hoard data, teams that burn out. Both biology and society suffer when regulation is mistaken for weakness. The immune system teaches that strength is measured by how precisely we activate under stress and how well we stand down when danger subsides. It reminds us that homeostasis is not constant activation, but the grace of returning to calm.

In biology, low−dose interleukin−2 selectively expands and stabilizes Tregs *in vivo*, showing signals of efficacy in chronic graft−versus−host disease and other settings (5, 6). Adoptive Treg therapy has graduated from concept to early trials, and engineering strategies (including CAR−Tregs) aim to grant antigen specificity, homing, and durability (7, 8). The frontier is not “more Tregs everywhere,” but precise control of when, where, and how Treg programs are deployed to induce localized, time−limited tolerance while preserving protective immunity. Precision, not power, defines equilibrium.

The sociology of Tregs is a parable for our laboratories and our communities. Claims became legible (CD25 as a handle), falsifiable (loss− and gain−of−function of FOXP3), and useful (bedside attempts to restore regulation) (1–5). Science, like immunity, depends on both curiosity and containment. Discovery without regulation burns out; regulation without curiosity stagnates.

## Conclusion

If Tregs were people, they would be essential workers who prevent the everyday from becoming the emergency. Honoring their discovery is honoring a principle: life (biological or civic) depends on our capacity to argue fiercely and then stand down. Regulation is not mere restraint; it is the quiet architecture that lets diverse cells, and diverse people, share a body. And perhaps that is the deepest wisdom of the immune system: that coexistence, not conquest, is the most sustainable form of survival.
